# Recurrent Episodes of Diffuse Alveolar Hemorrhage in Systemic Sclerosis 30 Days Apart

**DOI:** 10.1177/2324709619846594

**Published:** 2019-05-03

**Authors:** Hussien Saab, Tushar Bajaj, Kirandeep Bains, Ralph Garcia-Pacheco

**Affiliations:** 1 UCLA-Kern Medical, Bakersfield, CA, USA; 2 Ross University School of Medicine, Miramar, FL, USA

**Keywords:** critical care, pulmonary, hemorrhage, diffuse alveolar hemorrhage, systemic sclerosis, education

## Abstract

Diffuse alveolar hemorrhage (DAH) is a life-threatening clinicopathologic condition caused by accumulation of intra-alveolar red blood cells (RBCs) after disruption of the alveolar-capillary basement membrane that is often seen as a complication of various diseases, but is rare in systemic sclerosis. A 46-year-old female with systemic sclerosis presented to the emergency department complaining of right-sided chest pain. Initially, her electrocardiogram and chest X-ray (CXR) were unremarkable; however, she progressively decompensated into acute respiratory failure resulting in intubation. Repeat CXR and computed tomography scan showed diffuse bilateral alveolar infiltrates and pleural effusions. Video bronchoscopy with bronchoalveolar lavage showed numerous RBCs, neutrophils, macrophages, and respiratory epithelial cells consistent with acute DAH. She was started on intravenous pulse-dosing Solu-Medrol 1 g daily for 5 days. One month later, the patient returned with intractable nausea and vomiting. Again, she went into acute respiratory distress with a PaO_2_ of 59 while on a 10-L non-rebreather mask. CXR revealed development of alveolar infiltrates in the right lung. A bronchoscopy with bronchoalveolar lavage again showed numerous RBCs and neutrophils along with staining positive for hemosiderin-laden macrophages. Systemic sclerosis with alveolar hemorrhage is a rare occurrence; however, most cases are single episodes of hemorrhage, whereas we present a case with 2 confirmed episodes within 30 days. Its life-threatening nature makes a systemic approach and aggressive treatment crucial to decreasing morbidity and mortality—making it a diagnosis that should not be overlooked, especially in patients with nonspecific symptoms.

## Introduction

Diffuse alveolar hemorrhage (DAH) is a life-threatening clinicopathologic condition that results from an accumulation of intra-alveolar red blood cells after the disruption of the alveolar-capillary basement membrane. It can be associated with capillaritis, systemic vasculitis, or bland pulmonary hemorrhage, or it can be an isolated phenomenon with no associated processes or conditions.^1-3^ It is often seen as a complication in various pauci-immune or immune complex–associated diseases, such as systemic lupus erythematosus (SLE), but continues to be a rare occurrence in connective tissue diseases like systemic sclerosis. Here, we discuss a patient with known overlap syndrome, consisting of systemic sclerosis and dermatomyositis, who presented with 2 consecutive episodes of DAH within 30 days.

## Case Presentation

**Figure 1.**
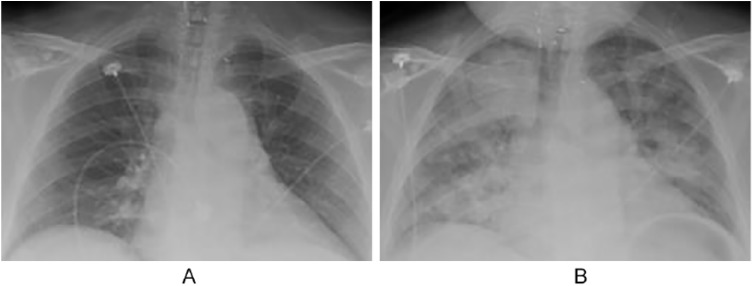
(A) Chest X-ray on admission. (B) Chest X-ray on hospital day 2 showing diffuse bilateral alveolar infiltrates.

**Figure 2.**
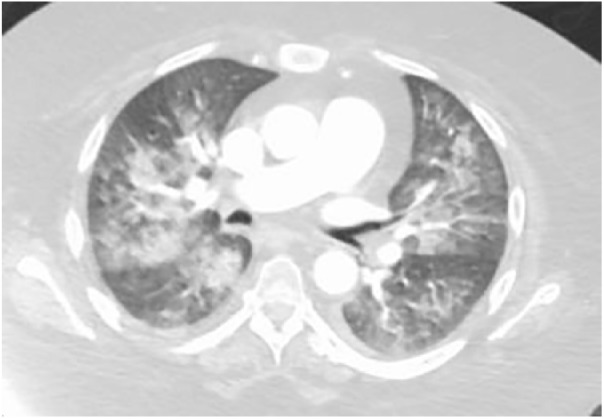
Computed tomography scan of chest with contrast with bilateral diffuse alveolar infiltrates and pleural effusions on hospital day 2.

A 46-year-old female with a medical history of systemic sclerosis that has features of dermatomyositis presented to the emergency department (ED) with complaints of right-sided chest pain. She recalled having similar pain in the past, which was attributed to scleroderma flare-ups. Physical examination demonstrated multiple cushingoid features secondary to her chronic steroid use, along with digit contractures and shiny, tight-appearing digits. On admission, her electrocardiogram and chest X-ray were unremarkable; however, the patient progressively decompensated and developed intractable nausea and vomiting. The patient subsequently went into acute respiratory failure with an arterial blood gas demonstrating a partial pressure of oxygen (PaO_2_) of 69 on 15 L of oxygen, resulting in intubation. Repeat chest X-ray and computed tomography showed diffuse bilateral alveolar infiltrates as well as bilateral pleural effusions (Figures 1 and 2). Video bronchoscopy with bronchoalveolar lavage (BAL) was performed that showed numerous red blood cells, neutrophils, macrophages, and respiratory epithelial cells consistent with acute DAH, but was negative for hemosiderin-laden macrophages.

After a Rheumatology consultation, the patient was started on intravenous (IV) pulse–dosing Solu-Medrol 1 g daily for 5 days, her azathioprine was increased to 75 mg, and her hydroxychloroquine was discontinued. Plasmapheresis was not deemed necessary at this time. Two days later, a repeat chest X-ray revealed partial clearing of the previous extensive bilateral alveolar densities. On improvement of symptoms, the patient was successfully extubated and later discharged. Her rheumatologist was contacted to ensure follow-ups for further management and starting the patient on IV immunoglobulin (IVIG) as outpatient.

**Figure 3.**
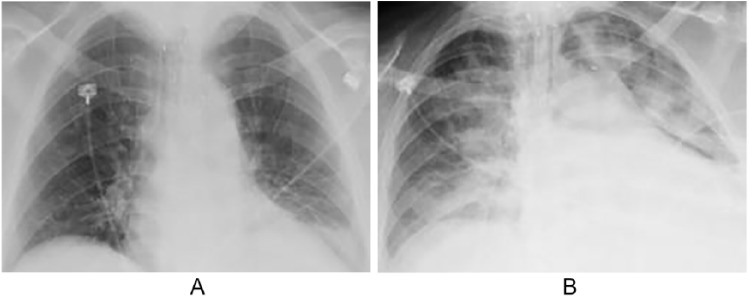
(A) Chest X-ray on admission. (B) Chest X-ray with diffuse alveolar infiltrates.

One month after discharge, the patient presented to the ED again, complaining of intractable nausea and vomiting. It was discovered that she had failed to start the outpatient IVIG therapy. During this admission, similar to the previous admission, the patient went into acute respiratory distress with a PaO_2_ of 59 on an arterial blood gas while on a 10-L non-rebreather mask. The chest X-ray at this time revealed interval development of alveolar infiltrates in the right lung (Figure 3). A bronchoscopy with BAL again showed numerous red blood cells and neutrophils along with staining positive for hemosiderin-laden macrophages, thus confirming her second episode of DAH. She was started on IVIG inpatient for her scleroderma crisis, which had to be discontinued after she developed tonic-clonic seizures shortly after the treatment. After stabilization, the patient was discharged with a follow-up with rheumatology for further management of her systemic sclerosis.

Since this last discharge, the patient has returned to the ED multiple times for cardiac and recurrent pyelonephritis episodes, but has not had a recurrent episode of DAH again.

## Discussion

Diffuse alveolar hemorrhage can have a myriad of underlying etiologies including systemic vasculitides, rheumatic disease, anti-glomerular basement membrane disease, idiopathic pulmonary hemosiderosis, and SLE; however, reports of DAH secondary to systemic sclerosis with alveolar hemorrhage are rare since the first description by Kallenbach et al in 1977.^2^ All of the previous reports encased a single episode of hemorrhage, whereas here we present a case with confirmed 2 episodes of DAH within 30 days.

Early recognition of DAH is vital for the patient’s outcome and highly dependent on its clinical, laboratory, radiologic, and pathologic features. DAH typically presents with the triad of hemoptysis, bilateral alveolar infiltrates on radiology, and hypoxemic respiratory failure; however, approximately 33% cases of DAH do not present with hemoptysis, as seen in our patient.^1,2,4^ Furthermore, DAH patients have been known to present with nonspecific symptoms of chest pain, cough, and dyspnea, as seen our patient, who presented with pleuritic chest pain accompanied with intractable vomiting and nausea. Chest X-rays can show nonspecific findings consisting of alveolar filling process that can be patchy, focal, or diffuse. Recurrent episodes of hemorrhage can lead to reticular interstitial opacities secondary to pulmonary fibrosis with mild degree of honeycombing. Computed tomography can give a more accurate picture with areas of consolidation, interspersed with areas of ground glass attenuation, and persevered normal areas.^1,2^

Moreover, early bronchoscopy with BAL is the preferred method to confirm the diagnosis of DAH and to rule out infectious causes. The diagnosis from BAL is confirmed when sequential lavage retrievals are progressively more hemorrhagic. To obtain a diagnosis of the underlying histopathology, a biopsy must be performed. The biopsy will help illustrate different histopathological patterns such as pulmonary capillaritis, bland pulmonary hemorrhage, or diffuse alveolar damage; however, it may not demonstrate the actual disease process leading to visualized histopathologic pattern. Staining for hemosiderin, which is the produce of hemoglobin degradation, appears at least 48 to 72 hours after bleeding and is useful to distinguish DAH from surgical trauma.^1-3^ Both bronchoalveolar lavages in our patient demonstrated progressive aliquots of hemorrhagic fluid, and the second presentation contained a biopsy, which stained positive for hemosiderin-laden macrophages.

Early recognition of DAH is crucial to implement the appropriate treatment for management. It has been noted that after hemorrhaging, patients develop severe progressive obstructive lung disease and emphysema due to the irreversible interstitial fibrosing from the alveolar damage, which is seen in many cases of SLE and microscopic polyangiitis.^2,4^ Appropriate treatment for DAH consists of addressing not only the autoimmune destruction of the alveolar-capillary basement membrane but also the underlying condition. Due to its association with a certain subset of conditions, corticosteroids and immunosuppressive agents remain the gold standard. Many experts recommend IV methylprednisolone at up to 500 mg every 6 hours for 4 to 5 days followed by gradual taper, as was done with this patient, who responded appropriately.^1,3^ Recombinant-activated human factor VII continues to be promising, but requires further evaluation.^1^

## Conclusion

Diffuse alveolar hemorrhages are a rare phenomenon in systemic sclerosis, let alone a recurrence of such episodes in the same patient within a short period of time, as was seen in our patient. Its life-threatening nature makes a systemic approach and aggressive treatment crucial to decreasing its associated morbidity and mortality, making it a diagnosis that should not be overlooked, especially in patient presenting with nonspecific symptoms.
